# A Novel Protocol for the Isolation of Fungal Extracellular Vesicles Reveals the Participation of a Putative Scramblase in Polysaccharide Export and Capsule Construction in *Cryptococcus gattii*

**DOI:** 10.1128/mSphere.00080-19

**Published:** 2019-03-20

**Authors:** Flavia C. G. Reis, Beatriz S. Borges, Luísa J. Jozefowicz, Bianca A. G. Sena, Ane W. A. Garcia, Lia C. Medeiros, Sharon T. Martins, Leandro Honorato, Augusto Schrank, Marilene H. Vainstein, Livia Kmetzsch, Leonardo Nimrichter, Lysangela R. Alves, Charley C. Staats, Marcio L. Rodrigues

**Affiliations:** a Instituto Carlos Chagas, Fundação Oswaldo Cruz (Fiocruz), Curitiba, Brazil; b Centro de Desenvolvimento Tecnológico em Saúde (CDTS), Fundação Oswaldo Cruz, Rio de Janeiro, Brazil; c Centro de Biotecnologia and Departamento de Biologia Molecular e Biotecnologia, Universidade Federal do Rio Grande do Sul, Porto Alegre, Brazil; d Instituto de Microbiologia Paulo de Góes (IMPG), Universidade Federal do Rio de Janeiro, Rio de Janeiro, Brazil; Carnegie Mellon University

**Keywords:** *Cryptococcus*, extracellular vesicles, fungi, secretion, scramblase

## Abstract

Regular protocols for the isolation of fungal extracellular vesicles (EVs) are time-consuming, hard to reproduce, and produce low yields. In an attempt to improve the protocols used for EV isolation, we explored a model of vesicle production after growth of Cryptococcus gattii and Cryptococcus neoformans on solid media. Nanoparticle tracking analysis in combination with transmission electron microscopy revealed that C. gattii and C. neoformans produced EVs in solid media. The properties of cryptococcal vesicles varied according to the culture medium used and the EV-producing species. EV detection was reproduced with an acapsular mutant of C. neoformans, as well as with isolates of Candida albicans, Histoplasma capsulatum, and Saccharomyces cerevisiae. Cryptococcal EVs produced in solid media were biologically active and contained regular vesicular components, including the major polysaccharide glucuronoxylomannan (GXM) and RNA. Since the protocol had higher yields and was much faster than the regular methods used for the isolation of fungal EVs, we asked if it would be applicable to address fundamental questions related to cryptococcal secretion. On the basis that polysaccharide export in *Cryptococcus* requires highly organized membrane traffic culminating with EV release, we analyzed the participation of a putative scramblase (Aim25; CNBG_3981) in EV-mediated GXM export and capsule formation in C. gattii. EVs from a C. gattii *aim25*Δ strain differed from those obtained from wild-type (WT) cells in physical-chemical properties and cargo. In a model of surface coating of an acapsular cryptococcal strain with vesicular GXM, EVs obtained from the *aim25*Δ mutant were more efficiently used as a source of capsular polysaccharides. Lack of the Aim25 scramblase resulted in disorganized membranes and increased capsular dimensions. These results associate the description of a novel protocol for the isolation of fungal EVs with the identification of a previously unknown regulator of polysaccharide release.

**IMPORTANCE** Extracellular vesicles (EVs) are fundamental components of the physiology of cells from all kingdoms. In pathogenic fungi, they participate in important mechanisms of transfer of antifungal resistance and virulence, as well as in immune stimulation and prion transmission. However, studies on the functions of fungal EVs are still limited by the lack of efficient methods for isolation of these compartments. In this study, we developed an alternative protocol for isolation of fungal EVs and demonstrated an application of this new methodology in the study of the physiology of the fungal pathogen Cryptococcus gattii. Our results describe a fast and reliable method for the study of fungal EVs and reveal the participation of scramblase, a phospholipid-translocating enzyme, in secretory processes of C. gattii.

## Abstract (precis)

Extracellular vesicles (EVs) are fundamental components of the physiology of cells from all kingdoms. In pathogenic fungi, they participate in important mechanisms of transfer of antifungal resistance and virulence, as well as in immune stimulation and prion transmission. However, studies on the functions of fungal EVs are still limited by the lack of efficient methods for isolation of these compartments. In this study, we developed an alternative protocol for isolation of fungal EVs and demonstrated an application of this new methodology in the study of the physiology of the fungal pathogen Cryptococcus gattii. Our results describe a fast and reliable method for the study of fungal EVs and reveal the participation of scramblase, a phospholipid-translocating enzyme, in secretory processes of C. gattii.

## INTRODUCTION

Extracellular vesicles (EVs) are produced in all domains of life (1). In fungi, these structures were first isolated in the human pathogen Cryptococcus neoformans (2). EVs have been further described in at least 11 additional species, and their functions in fungi include molecular transport across the cell wall (2), induction of drug resistance (3), prion transmission (4, 5), delivery of virulence factors (6, 7), immunological stimulation (8–12), RNA export (13), transfer of virulence traits (14), and transkingdom communication followed by regulation of expression of virulence-related genes (15). Although it is now well recognized that EVs play multiple and essential roles in fungal physiology, many questions remain unanswered (16). For instance, it is still unknown what mechanisms are required for biogenesis of EVs. The roles of EVs, if any, during infection also remain indefinite. Finally, as with other infection models, EVs have been proposed as vaccine candidates to prevent fungal diseases (8), but methods for obtaining large amounts of EVs for animal immunization are still not available. Indeed, many of the unsolved questions about fungal EVs remain unanswered because of experimental limitations. For instance, it is well known by researchers in the fungal EV field that the standard protocols used for vesicle isolation are time-consuming (1 to 3 weeks) and produce very low yields (17). It is clear, therefore, that the improvement of protocols for EV isolation might solve major questions in the field.

EVs have been traditionally studied after their isolation from liquid cultures (17). However, physiological production of EVs clearly does not demand liquid matrices. For instance, EVs are now considered structural and functional components of the extracellular matrix in mammalian models (18). In this environment, they participate in matrix organization, regulation of cellular functions, and determination of the physical properties of different tissues (19). EVs produced in gelatinous matrices impact tissue regeneration, inflammation, and tumor progression (18, 19). With a few exceptions (e.g., blood and liquor during infection), fungal cells are distributed over solid or gelatinous matrices, including the soil, bark of trees, bird excreta, and tissues of plants, insects, and higher animals. Nevertheless, the production of fungal EVs in non-liquid matrices has not been explored so far, although it is reasonable to suppose that fungal cells might produce EVs in non-liquid matrices.

Cryptococcus neoformans and Cryptococcus gattii use fungal EVs to export virulence factors and to promote cell-to-cell communication (7, 14). Noticeably, the *Cryptococcus* model is one of the most laborious systems in which fungal EVs have been studied, due to the low yields of the protocols and massive contamination with supernatant polysaccharides. EV-mediated molecular export in *Cryptococcus* demands membrane mobility (20). In this context, phospholipid flippases and scramblases are essential for membrane curvature and plasticity in different compartments of the cell (21). The flippase activity of aminophospholipid transferase 1 (Apt1) was implicated in EV production in C. neoformans (22, 23). The role of scramblases, however, remained unknown.

In this study, we describe a novel protocol for fast and reliable isolation of fungal EVs from solid media, mostly using C. gattii as a model. EV isolation from solid fungal cultures revealed the participation of a putative scramblase in EV formation and surface architecture of C. gattii. These results reveal novel approaches and cellular regulators implicated in the study of the functions and general properties of fungal EVs.

## RESULTS

### Fungal EVs are produced in solid media.

**FIG 1:**
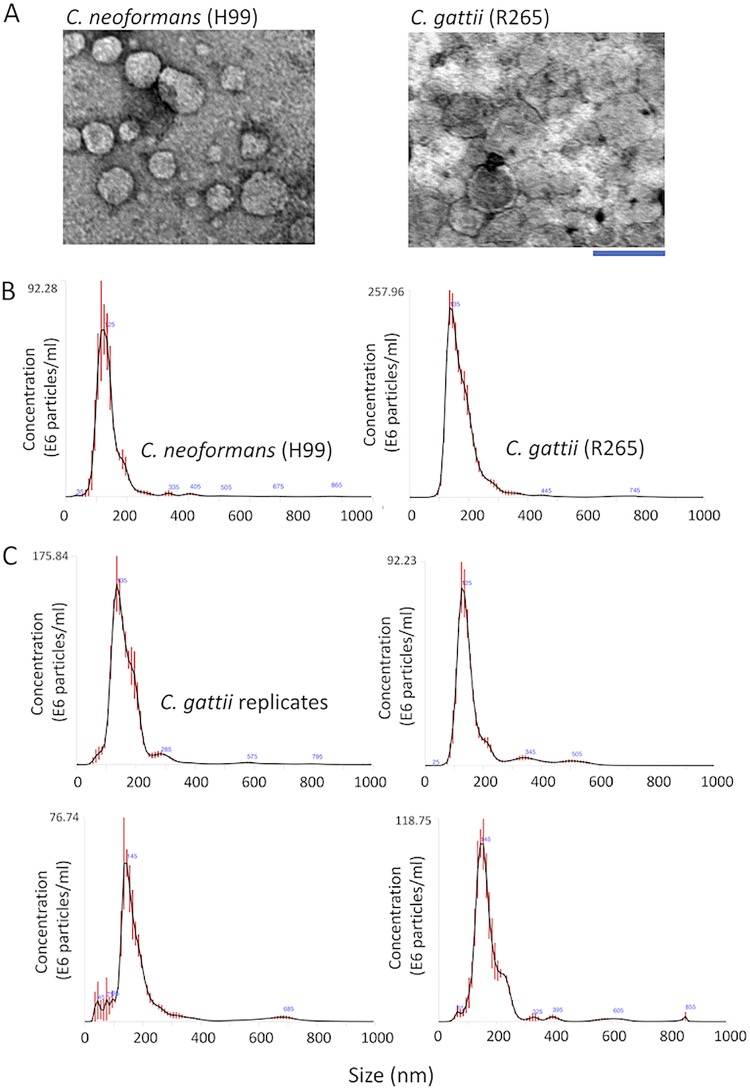
Isolation of fungal EVs from solid cultures of C. neoformans and C. gattii (strains H99 and R265, respectively). (A) Transmission electron microscopy of vesicular fractions obtained after growth of both pathogens on solid YPD. Scale bar, 200 nm. (B) Nanoparticle tracking analysis of the EV preparations illustrated in panel A, showing a concentration of vesicles in the range of 100 to 200 nm. (C) NTA profiles of four samples of C. gattii EVs obtained independently.

Due to the well-known limitations of the protocols currently used for the isolation of fungal EVs from liquid media (17), we asked whether these extracellular membrane compartments would be produced in solid matrices. We hypothesized that fungal EVs could be entrapped within the fungal population grown in plates containing solid media, which would favor a relatively high density of vesicles in an area of growth limited by the plate’s dimensions. To address this question, we cultivated C. gattii or C. neoformans cells to confluence in solid YPD (yeast extract-peptone-dextrose) for 24 h, for subsequent preparation of fungal suspensions in phosphate-buffered saline (PBS) after collection of fungal cells with inoculation loops (see Movie S1 in the supplemental material). Cell suspensions of 30 ml at densities varying from 5 × 10^9^ to 1 × 10^10^/ml were sequentially centrifuged to remove yeast cells and possible debris, and the remaining supernatants were ultracentrifuged to collect EVs. Ultracentrifugation pellets were negatively stained and analyzed by transmission electron microscopy (TEM), which revealed the presence of vesicular structures with the typical morphology and dimensions of fungal EVs (Fig. 1A). The same samples were submitted to nanoparticle tracking analysis (NTA), which revealed well-defined peaks corresponding to a major distribution of EVs within the size range of 100 to 300 nm (Fig. 1B). To analyze the reproducibility of the protocol, EV isolation from C. gattii was independently repeated four additional times, and vesicle properties were monitored by NTA. All samples produced very similar NTA profiles (Fig. 1C), indicating that the protocol was reproducible. Similar preparations were analyzed by dynamic light scattering, which has been consistently used for the analysis of EV dimensions (17). The profile of size distribution was similar to that obtained by NTA (data not shown).

10.1128/mSphere.00080-19.1 MOVIE S1 Preparation of fungal suspensions in PBS after collection of cells from solid media with inoculation loops. Download Movie S1, MOV file, 13.2 MB. Copyright © 2019 Reis et al. 2019 Reis et al. This content is distributed under the terms of the Creative Commons Attribution 4.0 International license.

**FIG 2:**
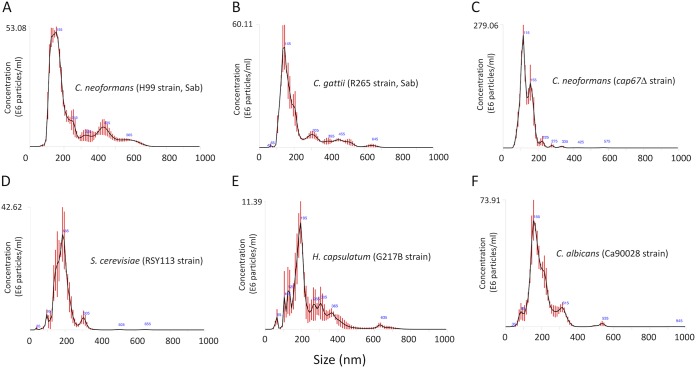
NTA profiles of EVs obtained from different fungal cultures in solid media. (A and B) Analysis of C. neoformans (A) and C. gattii (B) EVs obtained from Sabouraud cultures. (C) NTA of EVs obtained from an acapsular mutant of C. neoformans, suggesting that vesicle release in solid medium does not demand capsular structures. EVs were also detected by NTA after growth of S. cerevisiae (D), H. capsulatum (E), and C. albicans (F), indicating that the protocol is applicable to the study of different fungal pathogens.

We then asked if EV detection in solid media would only occur under specific experimental conditions. To address this question, we analyzed the production of EVs in a different medium or using distinct fungal species or strains. We first checked whether C. gattii and C. neoformans produced EVs in Sabouraud’s medium. Vesicular structures were abundantly detected, but the profile of size distribution included a minor population ranging from 300 to 600 nm in size (Fig. 2A and B). Since cryptococcal EVs have long been associated with the export of glucuronoxylomannan (GXM) (2), we also asked whether EV detection after growth in solid media would be influenced by the presence of the capsule. We then analyzed vesicles obtained from an acapsular mutant of C. neoformans. As revealed by NTA, the *cap67*Δ strain of C. neoformans also produced EVs (Fig. 2C). To investigate whether EV detection in solid media is exclusive to the *Cryptococcus* genus, we analyzed EV samples produced by Saccharomyces cerevisiae, Histoplasma capsulatum, and Candida albicans (Fig. 2D, E, and F). In all cultures tested, NTA revealed particles with properties that were compatible with EVs in size distribution. However, while C. albicans and S. cerevisiae gave nanoparticle signals that were similar to those found in C. neoformans and C. gattii, H. capsulatum produced EVs with a more diverse size distribution. Together, these results indicate that EV production in solid media is a general and consistently reproducible phenomenon.

### RNA and GXM are components of cryptococcal EVs produced in solid media.

**FIG 3:**
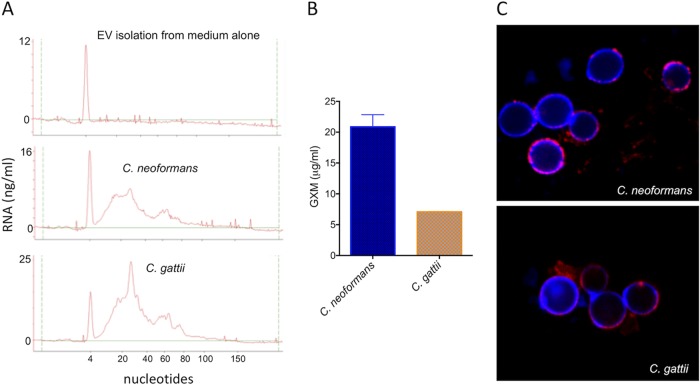
Analysis of the cargo of fungal EVs obtained from solid cultures of C. neoformans and C. gattii (strains H99 and R265, respectively). (A) Analysis of nucleic acid content confirmed the presence of small RNAs in cryptococcal vesicles. In these panels, the *y* axis corresponds to RNA detection as a function of fluorescence intensity, while the *x* axis represents RNA size in nucleotides. The first, sharp peak at 4 nucleotides corresponds to the RNA size marker. No RNA was detected in control samples obtained from the culture medium. (B) Detection of GXM in vesicular samples obtained from C. neoformans and C. gattii by ELISA. The concentration of vesicular GXM was significantly higher in C. neoformans samples (*P* = 0.0099). (C) Functional analysis of vesicular GXM in samples obtained from solid medium. The *cap67*Δ mutant of C. neoformans efficiently incorporated GXM (red fluorescence) from EVs produced by both C. neoformans and C. gattii in solid medium into the cell wall (blue fluorescence). Results in all panels are representative of three independent experiments.

Following the detection of EVs in solid cultures of fungal cells, we asked whether the cryptococcal membrane particles would contain the typical components that were previously described in fungal samples of EVs (2, 13). Since different RNA classes were previously characterized as components of EVs produced by C. neoformans, Malassezia sympodialis, Paracoccidioides brasiliensis, C. albicans, and Saccharomyces cerevisiae (13, 24), we investigated whether these nucleic acids were present in vesicle samples obtained from solid cultures of C. gattii and C. neoformans. Bioanalyzer analysis revealed the presence of RNA in EVs produced by both species (Fig. 3). The RNA pattern was similar to that observed for other eukaryotes, and most of the RNA was composed of molecules smaller than 200 nucleotides (nt), with a peak around 20 to 25 nt. The profiles of RNA detection were similar in both species (Fig. 3A).

GXM is another major component of cryptococcal EVs (2). We then analyzed C. neoformans and C. gattii samples for the presence of this polysaccharide by enzyme-linked immunosorbent assay (ELISA). EVs were disrupted by treatment with organic solvents, and GXM-containing precipitates were tested for reactivity with a monoclonal antibody to GXM (MAb 18B7), which confirmed the presence of the polysaccharide (Fig. 3B). The results differed in C. neoformans and C. gattii, with the latter showing a significantly smaller amount of vesicular GXM. These vesicular GXM samples were used in assays of polysaccharide incorporation into the surface of the *cap67*Δ acapsular mutant of C. neoformans. These cells efficiently incorporated the vesicular polysaccharide into their cell surface (Fig. 3C).

### A putative scramblase participates in EV-mediated export in C. gattii.

**FIG 4:**
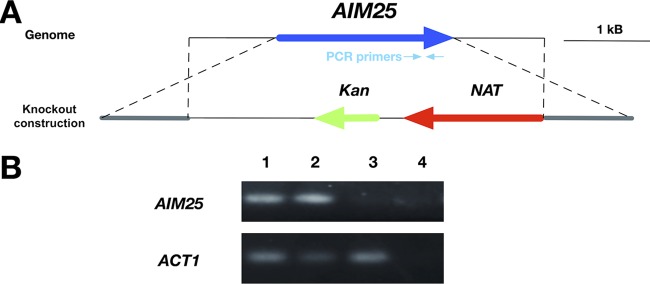
Construction of the *aim25*Δ mutant. (A) *AIM25* knockout scheme. The genome locus containing the *AIM25* gene and the knockout construct are shown in the upper and lower diagrams, respectively. NAT, cassette conferring nourseothricin resistance; Kan, cassette conferring kanamycin resistance for cloning purposes in Escherichia coli. Hybridization sites of the PCR primers are also shown. (B) Confirmation of *AIM25* deletion by PCR. Genomic DNA (100 ng) from WT cells (lane 1), a transformant with ectopic integration of the knockout cassette (lane 2) and the *aim25*Δ mutant (lane 3) was submitted to PCR using primers to amplify a segment of *AIM25* (upper panel) or the gene encoding action (*ACT1* [lower panel, loading control]). Control reactions without template addition are also shown (lane 4).

**FIG 5:**
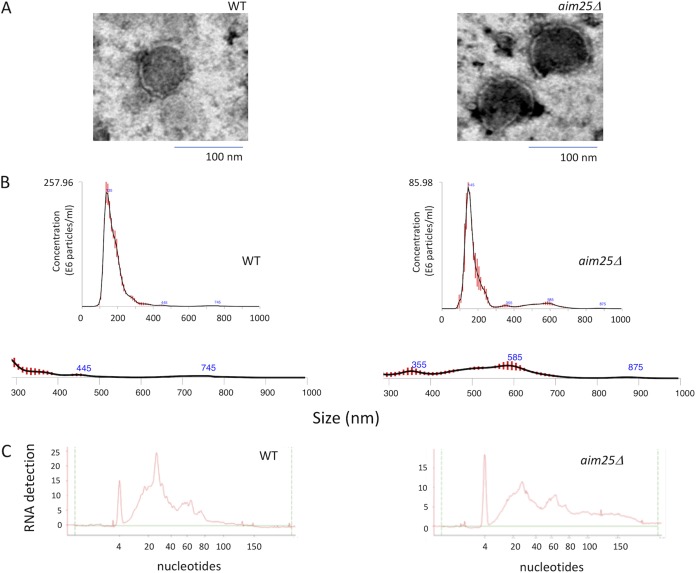
Analysis of EVs obtained after growth of wild-type (WT) or mutant (*aim25*Δ) cells of C. gattii in solid medium. (A) Transmission electron microscopy of WT and mutant cells. (B) NTA of EVs produced by WT and mutant cells, suggesting an increased detection of larger EVs (300 to 900 nm) in mutant cultures. The 300- to 900-nm size range of EVs was amplified below each NTA histogram. (C) Analysis of small RNAs contained in EVs produced by WT and mutant cells. The results shown in panels B and C are representative of two and three independent experiments, respectively.

On the basis of the consistent detection of EVs in solid cultures of C. neoformans and C. gattii, we asked whether the protocol would be applicable to address biological questions related to fungal secretion. On the basis of the role of eukaryotic scramblases in membrane traffic and secretion (21), we selected a putative scramblase that had been previously suggested in the C. gattii model as a regulator of secretion and target for antifungals (25) as a possible regulator of EV formation and/or polysaccharide release. The gene encoding the putative scramblase (*AIM25*; CNBG_3981) was knocked down in the R265 background of C. gattii (Fig. 4), and the resulting mutant cells were phenotypically characterized. The mutant had normal proliferation rates (not shown) and produced EVs in solid medium (Fig. 5A). The amounts of EVs produced by mutant cells tended to be smaller, but no statistical significance was observed (data not shown). Deletion of *AIM25*, however, resulted in the production of a population of EVs of larger dimensions, in comparison to vesicles produced by wild-type C. gattii (Fig. 5B). Vesicular components were also affected in the *aim25*Δ mutant, as concluded from the altered profile of RNA detection in mutant EVs (Fig. 5C).

**FIG 6:**
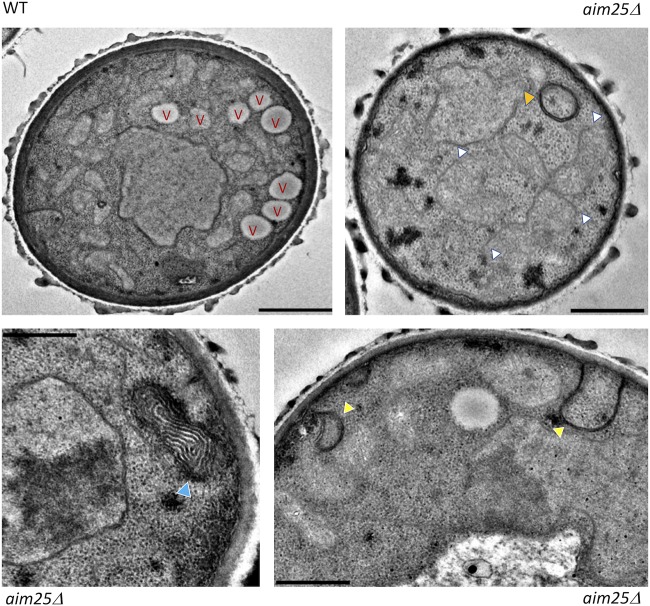
Transmission electron microscopy of wild-type (WT) and mutant (*aim25*Δ) cells of C. gattii. WT cells manifested the typical intracellular morphology of cryptococci, including well-defined vacuoles (V) and organized membranous compartments. In mutant cells, distorted membranes were abundantly detected. Phenotypic traits that were exclusive to mutant cells included a general lack of the typical cryptococcal vacuoles, highly-electron-dense membranous compartments (orange arrowhead), linearized membranes (white arrowheads), electron-dense, stacked membranes (blue arrowhead), and atypical invaginations of the plasma membrane (yellow arrowheads). Scale bars correspond to 200 nm.

The differences in EV dimensions and cargo were suggestive of a role of the putative scramblase in membrane organization and/or EV biogenesis. In fact, transmission electron microscopy (TEM) revealed that C. gattii *aim25*Δ mutant cells had clearly disorganized membranes, which included a general lack of the typical cryptococcal vacuoles, aberrant membranous structures, atypical plasma membrane invaginations, and linearized membranous filaments with no apparent connections with cellular organelles (Fig. 6). These results were consistent with the primary roles played by scramblases in the membrane organization of other eukaryotic cells (21).

**FIG 7:**
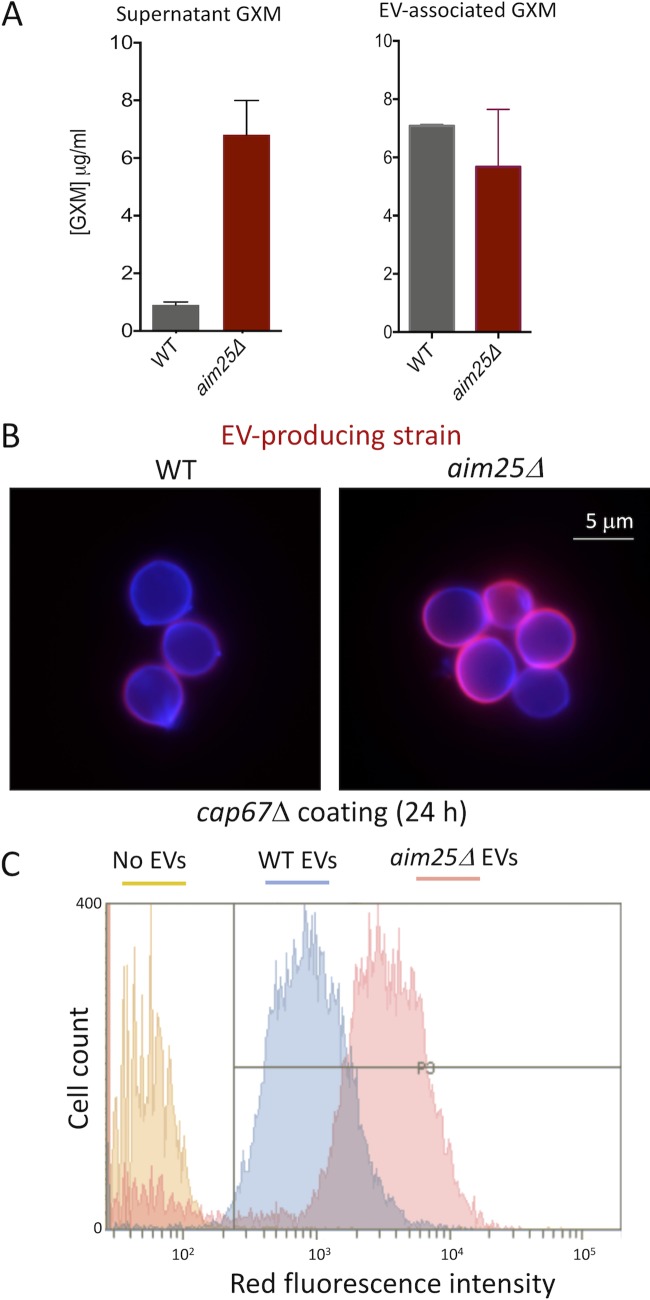
Analysis of extracellular GXM in wild-type (WT) and mutant (*aim25*Δ) cells of C. gattii. (A) Determination of extracellular GXM in supernatant samples (A) demonstrated that mutant cells produced significantly increased polysaccharide concentrations (*P* = 0.0001). No significant changes in the GXM content (*P* = 0.41) were observed in EV samples. (B) Microscopic examination of the ability of *cap67*Δ cells to incorporate GXM obtained from C. gattii suggested that GXM incorporation by the acapsular strain was more efficient when *aim25*Δ vesicles were used. Blue fluorescence denotes cell wall staining with calcofluor white. Red fluorescence corresponds to GXM staining with MAb 18B7. (C) Flow cytometry analysis of acapsular cells under the conditions described in panel B, providing a quantitative confirmation of the visual observation resulting from microscopic analysis. Results are representative of two independent experiments.

Polysaccharide export in *Cryptococcus* relies on membrane mobility and vesicular traffic (2, 20). Therefore, we quantified GXM in crude supernatant samples and EV fractions obtained from wild-type and mutant cells of C. gattii. In comparison with parental cells, the concentration of GXM was much higher in the supernatants of the *aim25*Δ mutant strain (Fig. 7A). However, no significant differences were observed when GXM was quantified in vesicular fractions, although the mutant tended to produce reduced amounts of EV-associated GXM. We therefore hypothesized that in the absence of scramblase, GXM could be more efficiently released from EVs, becoming more abundant in soluble supernatant fractions. To address this question, we compared the ability of acapsular *cap67*Δ cells to incorporate vesicle-associated polysaccharide obtained from wild-type cells and the *aim25*Δ mutant. After 24 h of incubation, the acapsular *cap67*Δ cells incorporated GXM from EVs produced by the *aim25*Δ scramblase mutant more efficiently than from wild-type vesicles, as concluded by immunofluorescence (Fig. 7B) and flow cytometry (Fig. 7C) analyses. This result agrees with a more efficient extraction of GXM from vesicles obtained by fungal cells lacking *AIM25*.

**FIG 8:**
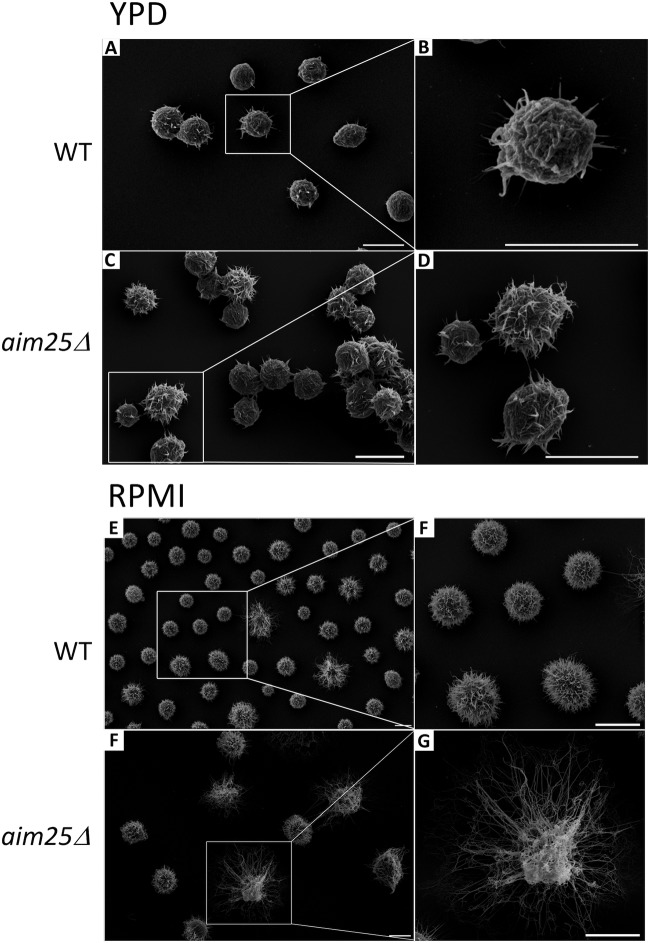
Scanning electron microscopy of wild-type (WT) and mutant (*aim25*Δ) cells of C. gattii after growth in solid YPD (capsule repression) or incubation in RPMI (capsule induction). General views of WT (A and E) or *aim25*Δ (C and F) cells are shown for each condition in the left panels. Magnified views of WT (B and F) or *aim25*Δ (D and G) cells from the insets in the left panels are shown in the right panels. Scale bars correspond to 5 μm.

**FIG 9:**
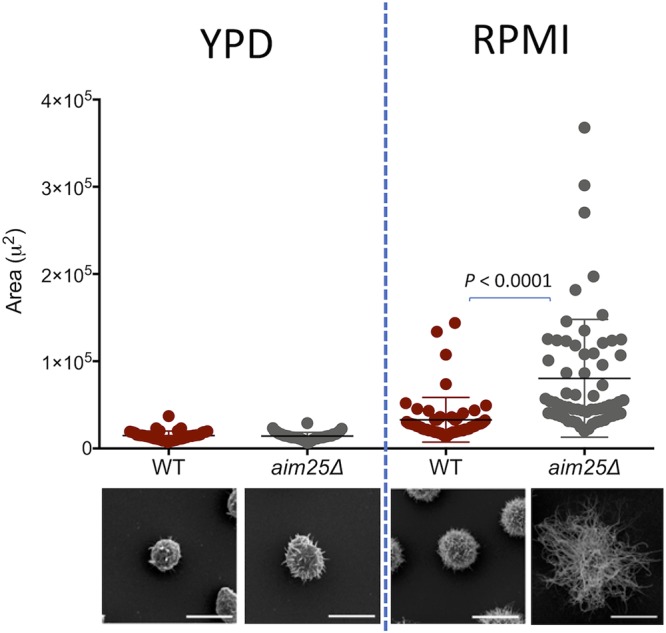
Analysis of cellular area as a consequence of capsular dimensions in wild-type (WT) and mutant (*aim25*Δ) cells of C. gattii after growth in solid YPD (capsule repression) or incubation in RPMI (capsule induction). The most representative phenotypes observed under each experimental condition are shown on the bottom as scanning electron microscopy images. Differences in capsular dimensions had no statistical significance, with the exception of the comparison between WT and mutant cells after incubation in RPMI. Scale bars correspond to 5 μm.

Since GXM export in EVs and increased concentration of capsular polysaccharides in supernatants were linked to capsular enlargement (2), we asked if the *aim25*Δ mutant was more efficient in producing large capsules than wild-type cells of C. gattii. Wild-type and mutant cells had their capsular morphology first analyzed by India ink counterstaining, and the results suggested that deletion of *AIM25* led to increased capsular dimensions (data not shown). For a more detailed analysis of the dimensions and morphology of capsule fibers, scanning electron microscopy was performed. We first analyzed fungal cells under the conditions of EV isolation. Growth in YPD inhibits capsule formation (26), and as expected, capsular dimensions were reduced in both wild-type and *aim25*Δ mutant cells cultivated in the solid medium (Fig. 8). A closer analysis of fungal cells, however, indicated that capsule fibers, although small in dimension, were more abundant in mutant cells grown in YPD (Fig. 8A to D). We then asked whether eventual differences in capsular structures would become more evident under conditions of capsule induction in RPMI (26). This approach in fact resulted in fungal cells with larger capsules (Fig. 8E to G). Under these conditions, yeast cells with more exuberant capsules were apparently more frequently observed in *aim25*Δ mutant populations. To confirm the visual perception that capsule enlargement was facilitated in mutant cells, we quantified the average capsular dimensions under conditions of capsule repression (YPD) and induction (RPMI) (Fig. 9). Despite the increased number of capsular fibers in mutant cells, no differences in capsular dimensions were observed after growth of C. gattii in YPD. Capsule induction in RPMI, however, was significantly more efficient in mutant cells. Together, these results indicate that deletion of *AIM25* resulted in a more efficient extracellular release of GXM, resulting in facilitated capsule enlargement.

## DISCUSSION

Several aspects of the biology of fungal EVs remain to be elucidated (16), and the reduced knowledge on the characteristics of fungal EVs is likely a consequence of inefficient protocols, which usually involve centrifugation of liters of cultures, massive loss of biologically active samples, and very low yields (17). Fungal pathogens are rarely found in liquid matrices. Except for the cases of fungemia and liquor contamination, pathogenic fungi are usually colonizing tissues, mucosae, and the extracellular matrix during infection (27). In the environment, fungal species with pathogenic potential to humans and animals are usually distributed into the soil, tree shelves, and animal excreta (27). In this context, we hypothesized that fungal EVs could be produced in solid matrices. Besides the biological aspects of EV production in solid media, isolation of vesicle preparations would be facilitated by easier control of both area and volume limitation imposed by flasks or plates containing solid medium. We therefore designed a protocol through which extracellular fungal components would be collected from solid media and suspended in relatively reduced volumes for further ultracentrifugation. This protocol was efficient for different fungal species, highly reproducible, and essentially fast. EVs isolated from solid media were shown to be biologically active and to contain at least some of the typical components of EVs, as concluded from experiments demonstrating the traffic of GXM and presence of RNA, respectively.

Traditional protocols of EV isolation from cryptococcal cultures usually include massive GXM contamination (17). However, it is still premature to affirm that this issue was resolved with the current protocol, since the culture conditions used in this study for EV isolation from solid media are known to inhibit GXM secretion and capsule formation (26). Importantly, while the protocols available for isolation of fungal EVs could take up to weeks and include numerous rounds of supernatant concentration and ultracentrifugation (17), the proposed protocol took approximately 5 h from collection of extracellular components to NTA analysis without any additional cost. We also showed that the facilitated protocol for EV isolation was applicable to address important biological questions related to fungal export. For example, our study compared for the first time the properties of EVs produced by C. neoformans and C. gattii, which revealed important differences in polysaccharide content.

We extended the currently described approach to the study of potential regulators of EV-mediated export. Scramblases and flippases are different types of enzymatic groups of phospholipid transportation enzymes (21). It is reasonable to suppose that regulators of membrane architecture are required for proper EV release. Indeed, in C. neoformans, the Apt1 flippase regulated EV physical properties and GXM export (22, 23). The participation of other membrane regulators remained unexplored, as well as the role of phospholipid translocators in the C. gattii model. In this context, we selected a putative scramblase (Aim25) as a potential regulator of membrane architecture and EV formation in the C. gattii model.

Both WT and *aim25*Δ mutant cells lacking the gene putatively encoding the C. gattii scramblase produced EVs. Based on the detection of a population of larger EVs in cultures of mutant cells, we hypothesized that scramblase was required for membrane organization, proper EV formation, and extracellular cargo release from fungal cells. Membrane organization and proper EV formation were in fact affected in *aim25*Δ mutant cells, as concluded from TEM and NTA analyses. EV cargo was likely impacted in mutant cells, as concluded from the altered profile of RNA detection in mutant vesicles. Unexpectedly, the GXM concentration was highly increased in whole supernatants of *aim25*Δ mutant cells. We therefore hypothesized that the altered membrane organization of mutant cells could result in a more efficient release of GXM from EVs. If this hypothesis was valid, a more efficient capsule formation would be expected in *aim25*Δ mutant cells. Our results demonstrated that *cap67*Δ acapsular cells were indeed more efficient in taking up GXM from EVs produced by the *aim25*Δ mutant. In fact, these scramblase mutant cells had more exuberant capsules. These results contradict the notion that deletion of membrane regulators will negatively impact GXM export and capsule formation and efficiently illustrate the complexity of the physiological functions of EV-mediated molecular export.

The pathogenic potential of the *aim25*Δ mutant still needs to be addressed, but the impact of scramblase deletion in *Cryptococcus* is likely broader. For instance, mutant cells of C. neoformans lacking flippase expression had aberrant Golgi structure, attenuated synthesis of phospholipids, increased production of immunogenic sterols, and reduced formation of virulence-related lipids (22, 23). Flippase and scramblase functions, however, are not necessarily related. In contrast to our current observations, flippase mutants had decreased GXM synthesis and reduced capsular dimensions (22).

Our present results describe a novel, simplified protocol of EV isolation and its application to reveal functions of a previously unknown regulator (Aim25 scramblase) of EV properties in *Cryptococcus*. The impact of the use of the new approach to study fungal EVs will be revealed in the future, but considering the well-known difficulties in the field, it is expected that the protocol will be useful not only to identify other regulators of EV formation, but also to address the immunological functions of fungal EVs and to develop new alternatives to the study of their biogenesis and composition. The potential of this methodology to investigate EV formation in different fungal species and morphological stages is also foreseeable.

## MATERIALS AND METHODS

### Fungal strains.

The EV-producing isolates used in this study included the standard strains H99 of C. neoformans and R265 of C. gattii (2, 14), C. albicans strain ATCC 90028 (8), S. cerevisiae strain RSY113 (28), and H. capsulatum strain G217B (29). The *cap67*Δ acapsular mutant of C. neoformans was used for glycan incorporation assays (30). The Delsgate methodology was used to construct the *aim25*Δ mutant strain lacking scramblase expression in the C. gattii background (CNBG_3981 in C. gattii R265; ortholog CNAG_07164 in C. neoformans H99). Two fragments (∼1,000 bp) encompassing the 5′ and 3′ flanking sequences of the CNBG_3981 locus were PCR amplified and gel purified using the PureLink Quick Gel extraction and PCR purification combo kit (Invitrogen). Both fragments were mixed (100 ng of each) with pDONR-NAT vector (∼200 ng), as previously described (31), and submitted to BP clonase reaction according to manufacturer’s instructions (Invitrogen). The cassette was transformed in Escherichia coli OmniMAX cells and selected by antibiotic resistance screening and colony PCR. Biolistic transformation was performed to introduce the deletion construct previously linearized by I-SceI enzymatic digestion in C. gattii, as previously described (31). The screening was performed using nourseothricin resistance and colony PCR. The mutant strain was confirmed by semiquantitative reverse transcription-PCR (RT-PCR) using actin transcripts as a loading control, according to protocols previously used (31). The primers used are listed in Table 1. Stock cultures of C. neoformans, C. gattii, S. cerevisiae, and C. albicans were maintained through passages in Sabouraud plates. H. capsulatum was kept in brain heart infusion agar supplemented with sheep blood (5%).

**TABLE 1:** Primers used for deletion of a putative scramblase (Aim25; CNBG_3981) of C. gattii

Primer	Sequence (5′→3′)	Purpose
RT-CNBG_3981F	TTTGGAAGGGTATGAGGAAGAG	RT-PCR of CNBG_3981
RT-CNBG_3981R	ACTACCTCCACCAAACCAAC	RT-PCR of CNBG_3981
Actin_F	CGGTATCGTCACAAACTGG	RT-PCR of actin
Actin_R	GGAGCCTCGGTAAGAAGAAC	RT-PCR of actin
CNBG3981_5F	AAAATAGGGATAACAGGG TAATCCCTTGATGCTTCCTC TCATC	Amplification of 5′ flanking region for deletion construct
CNBG3981_5R	GGGACAAGTTTGTACAAAA AAGCAGGCTATGTAAGACG GACGGTTGTTAGAG	Amplification of 5′ flanking region for deletion construct
CNBG3981_3F	GGGGACCACTTTGTACAAG AAAGCTGGGTAGCCTTGGG CTATGTGAAATC	Amplification of 3′ flanking region for deletion construct
CNBG3981_3R	AAAAATTACCCTGTTATCC CTAGGGCTAATGCGAGTTG TAAAG	Amplification of 3′ flanking region for deletion construct

### EV isolation from solid media.

One colony of each isolate cultivated in solid Sabouraud’s medium was inoculated into 5 ml of yeast extract-peptone-dextrose (YPD) medium and cultivated for 2 days at 30°C with shaking. Due to specific nutritional requirements, the only exception was H. capsulatum, which was cultivated in Ham’s F-12 medium for 48 h at 37°C with shaking. The cells were counted and diluted to a density of 3.5× 10^7^cells/ml in YPD. Aliquots of 300 μl of these cell suspensions were spread onto YPD agar plates (90- by 15-mm petri dishes containing 25 ml of medium) and incubated for 1 day at 30°C to reach confluence. Once again, the exception was H. capsulatum, which was cultivated in Ham’s F-12 agar and incubated for 48 h at 37°C.Three petri dishes were used for each EV isolation. The cells were gently recovered from each of the three plates with an inoculation loop and transferred to a single centrifuge tube containing 30 ml of PBS (Movie S1) previously sterilized by filtration through 0.22-μm-pore membranes. Suspended cells were collected by centrifugation at 5,000 × *g* for 15 min at 4°C. The supernatants were collected and centrifuged again at 15,000 × *g* for 15 min at 4°C to remove debris. The resulting supernatants were filtered through 0.45-μm-pore syringe filters and centrifuged at 100,000 × *g* for 1 h at 4°C. Supernatants were discarded and pellets suspended in 300 μl of sterile PBS. EV preparations were maintained at 4°C. The presence of EVs was monitored by nanoparticle tracking analysis and electron microscopy, as detailed below.

### Transmission electron microscopy.

Fungal cells were washed twice in PBS and fixed for 1 h in 2.5% glutaraldehyde in 0.1 M phosphate buffer at room temperature. The fixed yeast cells were washed twice in 0.1 M cacodylate buffer and then postfixed with 1% osmium tetroxide–1.6% potassium ferrocyanide–5 mM CaCl_2_ diluted in 0.1 M cacodylate buffer for 30 min at room temperature. The samples were washed three times with 0.1 M cacodylate buffer, dehydrated in a graded acetone series (5 min at 30, 50, 70, 90, and 100%), and then embedded in PolyBed812 resin. Ultrathin sections were obtained in a Leica EM UC6 ultramicrotome, collected on copper grids, contrasted with 5% uranyl acetate and lead citrate, and then visualized in a JEOL 1400Plus transmission electron microscope at 90 kV. For negative-stain electron microscopy of EVs, samples obtained from solid media were transferred to carbon- and Formvar-coated grids and negatively stained with 1 % uranyl acetate for 10 min. The grids were then blotted dry before immediately being observed in a JEOL 1400Plus transmission electron microscope at 90 kV.

### NTA.

NTA of fungal EVs was performed on an LM10 nanoparticle analysis system, coupled with a 488-nm laser and equipped with an _S_CMOS camera and a syringe pump (Malvern Panalytical, Malvern, United Kingdom), as recently described for cryptococcal EVs (32). All samples were 20- to 50-fold diluted in filtered PBS and measured within the optimal dilution range of 9 × 10^7^ to 2.9 × 10^9^ particles/ml. Samples were injected using a syringe pump speed of 50, and three videos of 60 s were captured per sample, with the camera level set to 15, gain set to 3, and viscosity set to that of water (0.954 to 0.955 cP). For data analysis, the gain was set to 10 to 15 and the detection threshold was set to 2 to 3 for all samples. Levels of blur and maximum jump distance were automatically set. The data were acquired and analyzed using the NTA 3.0 software (Malvern Panalytical).

### RNA isolation and analysis.

Vesicular RNA was obtained as previously described by our group (13, 32) with the mirCURYTM RNA isolation kit (Qiagen), used according to the manufacturer’s instructions. As a control, we performed RNA isolation from the solid medium alone, which gave negative results. For quantitative determination, RNA samples were analyzed with an RNA Agilent 2100 Bioanalyzer (Agilent Technologies) set up for detection of small RNA (sRNA) molecules, as described in recent studies by our group (13, 32). Comparisons between wild-type and mutant cells demanded normalization to the number of vesicles in each sample.

### Analysis of extracellular GXM.

The presence of GXM in crude supernatant fractions was analyzed by ELISA as previously described (33). Standard solutions of GXM were prepared after polysaccharide aggregation by ultrafiltration of supernatants as previously established by our group (34). For GXM quantification in EV fractions, aliquots of 7.8 × 10^8^ EV particles were vacuum dried and disrupted by the addition of 100 μl of a mixture of chloroform and methanol (1:2 [vol/vol]). Precipitated polysaccharides were obtained by pulse centrifugation and subsequently delipidated by similar rounds of precipitation using other mixtures of chloroform and methanol (2:1 and 9:1 at 100 μl each). The dried precipitates were suspended in PBS (50 μl) for GXM quantification by ELISA (33). Comparisons between wild-type and mutant cells demanded normalization to the number of vesicles in each sample.

### Incorporation of GXM into the surface of acapsular cells.

Acapsular C. neoformans cells (*cap67*Δ strain) cells were grown in YPD for 24 h, at 30°C with shaking (200 rpm). Yeast cells (5 × 10^6^ cells) were collected by centrifugation and washed twice in PBS. The cells were then suspended in 150 μl PBS containing 20- to 50-fold diluted 8 × 10^8^ EVs (particle number estimated by NTA) or GXM precipitated from the same particle number and incubated at room temperature for 24 h. After incubation, the cells were extensively washed with PBS and processed for immunofluorescence as previously described by our group (35). In these assays, the cell wall was stained in blue with calcofluor white and capsular structures appeared in red, after incubation with MAb 18B7. The cells were visualized on a Leica TCS SP5 confocal microscope or analyzed with a FACS Canto II flow cytometer. Data were processed with the FACSDiva software, version 6.1.3.

### Scanning electron microscopy.

Fungal cells were grown on solid YPD as described before (under capsule repression conditions) or incubated in RPMI for capsule induction. For capsule enlargement, 2 × 10^6^ cells were suspended in 200 μl of RPMI and incubated for 24 h at 37°C under a 5% CO_2_ atmosphere. Cryptococcal cells were washed three times with PBS and fixed in 2.5% glutaraldehyde in 0.1 M sodium cacodylate buffer (pH 7.2) for 1 h at room temperature. The cells were then washed three times with 0.1 M sodium cacodylate buffer (pH 7.2) containing 0.2 M sucrose and 2 mM MgCl_2_. Washed cells were adhered to coverslips that were previously coated with 0.01% poly-l-lysine (Sigma-Aldrich) for 20 min. Adhered cells were gradually dehydrated in ethanol (30, 50, and 70% for 5 min and then 95% and 100% twice for 10 min). The samples were critical point dried (Leica EM CPD300) immediately after dehydration, mounted on metallic bases (stubs), coated with a gold layer of 15- to 20-nm particles (Leica EM ACE200), and finally visualized in a scanning electron microscope (JEOL JSM-6010 Plus/LA) operating at 20 kV. For analysis of capsular dimensions, at least 50 cells were analyzed individually and had their total area determined using ImageJ software (National Institutes of Health). Since no differences in cell bodies were observed between the different strains and conditions used in this study, we assumed that differences in the total cellular area reflected alterations in capsular dimensions.

### Statistics.

Statistical analyses were performed with the GraphPad software (La Jolla, CA). Group comparisons were submitted to one-way analysis of variance (ANOVA) followed by the Tukey’s multiple-comparison test.
